# Cytogenetics in Chronic Lymphocytic Leukemia: ERIC Perspectives and Recommendations

**DOI:** 10.1097/HS9.0000000000000707

**Published:** 2022-03-25

**Authors:** Panagiotis Baliakas, Blanca Espinet, Clemens Mellink, Marie Jarosova, Anastasia Athanasiadou, Paolo Ghia, Arnon P. Kater, David Oscier, Claudia Haferlach, Kostas Stamatopoulos

**Affiliations:** 1 Department of Immunology, Genetics and Pathology, Science for Life Laboratory, Uppsala University, Sweden; 2 Department of Clinical Genetics, Uppsala University Hospital, Sweden; 3 Molecular Cytogenetics Laboratory, Pathology Department, Hospital del Mar, Barcelona, Spain; 4 Translational Research on Hematological Neoplasms Group, Cancer Research Program, Institut Hospital del Mar d’Investigacions Mèdiques (IMIM), Barcelona, Spain; 5 Genomics Laboratory, Department of Clinical Genetics, Amsterdam University Medical Centers, University of Amsterdam, the Netherlands; 6 Institute of Medical Genetics and Genomics, Faculty of Medicine, Masaryk University and University Hospital Brno, Czech Republic; 7 Department of Internal Medicine - Hematology and Oncology, University Hospital Brno and Faculty of Medicine, Masaryk University, Brno, Czech Republic; 8 Hematology Department and HCT Unit, G. Papanicolaou Hospital, Thessaloniki, Greece; 9 Strategic Research Program in CLL and BCell Neoplasia Unit, Division of Experimental Oncology, IRCCS Ospedale San Raffaele and Università Vita-Salute San Raffaele, Milan, Italy; 10 Department of Hematology, Cancer Center Amsterdam, Lymphoma and Myeloma Center Amsterdam, Amsterdam University Medical Centers, University of Amsterdam, the Netherlands; 11 Department of Haematology, Royal Bournemouth Hospital, United Kingdom; 12 MLL Munich Leukemia Laboratory, Munich, Germany; 13 Institute of Applied Biosciences, Center for Research and Technology Hellas, Thessaloniki, Greece; 14 Department of Molecular Medicine and Surgery, Karolinska Institutet, Stockholm, Sweden

## Abstract

Mounting evidence underscores the clinical value of cytogenetic analysis in chronic lymphocytic leukemia (CLL), particularly as it allows the identification of complex karyotype, that has recently emerged as a prognostic and potentially predictive biomarker. That said, explicit recommendations regarding the methodology and clinical interpretation of either chromosome banding analysis (CBA) or chromosome microarray analysis (CMA) are still lacking. We herein present the consensus of the Cytogenetic Steering Scientific Committee of ERIC, the European Research Initiative on CLL, regarding methodological issues as well as clinical interpretation of CBA/CMA and discuss their relevance in CLL. ERIC considers CBA standardized and feasible for CLL on the condition that standards are met, extending from the use of novel mitogens to the accurate interpretation of the findings. On the other hand, CMA, is also standardized, however, robust data on its clinical utility are still scarce. In conclusion, cytogenetic analysis is not yet mature enough to guide treatment choices in CLL. That notwithstanding, ERIC encourages the wide application of CBA, and potentially also CMA, in clinical trials in order to obtain robust evidence regarding the predictive value of specific cytogenetic profiles towards refining risk stratification and improving the management of patients with CLL.

## CYTOGENETIC ANALYSIS IN CHRONIC LYMPHOCYTIC LEUKEMIA: A HISTORICAL OVERVIEW

The first reports on chromosome banding analysis (CBA) in chronic lymphocytic leukemia (CLL) were published in the 1980s. Metaphases were successfully obtained in 40% to 50% of cases, 40% to 50% of them harboring at least one cytogenetic aberration.^1^ In the ensuing years, improved cultivation, including stimulation with B-cell mitogens led to a success rate to obtain metaphases of 90% and an aberration incidence of ~60% to 70%.^2^ In contrast to acute leukemias and myelodysplastic syndromes, CBA was not introduced into routine diagnostics at that time. The reasons were the still low in vitro proliferative capacity of CLL cells resulting in missing the abnormal CLL clone in a substantial fraction of cases, as revealed by array comparative genomic hybridization (CGH) and interphase fluorescence in situ hybridization (FISH) data.^3,4^

The hallmark paper on interphase FISH in CLL was published in 2000.^5^ With the help of only 4 probes targeting deletions of the 13q14, 11q22 (*ATM*), 17p13 (*TP53*) regions, and trisomy 12, cytogenetic abnormalities were detected in the great majority of cases and the prognostic value of interphase FISH in CLL was demonstrated^5^: thus, interphase FISH became the cytogenetic technique of choice for the characterization of CLL. This was reflected in the guidelines for the diagnosis and treatment of CLL published by the International Workshop on Chronic Lymphocytic Leukemia (iwCLL) in 2008 which recommended to perform interphase FISH always in clinical trials and suggested to perform it also in clinical practice before every new line of treatment.^6^

In parallel, the application of specific culturing protocols, particularly with the usage of specific mitogens, namely CD40 ligand (CD40L) or CpG-oligonucleotide DSP30 plus interleukin-2 (IL-2)^2,7^ reached rates of aberrant karyotypes comparable to those obtained by FISH.^8,9^ Importantly, the usage of CpG/IL-2 did not induce any clonal cytogenetic changes,^8,10^ hence, the detected abnormalities represent true CLL-associated aberrations.

Studies using both CBA and interphase FISH in parallel revealed that these techniques complement each other.^11^ In more detail, in cases with normal FISH, CBA detected abnormalities that were not covered by the panel of FISH probes used.^10,12^ On the other hand, FISH detected abnormalities that were not identified by CBA due to its inherently lower resolution. Indicatively, the majority of 13q deletions in CLL are too small to be visible by CBA but can be detected by interphase FISH. Moreover, aberrations with potential clinical significance such as deletion of 6q21, gain of 2p, recurrent translocations [e.g. t(14;19)] or complex karyotypes (CK) are visible with CBA but not detected with the standard CLL FISH analysis.^12–15^ Thus, performing CBA in combination with interphase FISH results in a more comprehensive genetic characterization of CLL.

Similarly to FISH, the application of chromosome microarray analysis (CMA) in CLL has been associated with increasing detection of cytogenetic abnormalities with potential clinical significance. Genomic array platforms such as array-based CGH (array-CGH) and single nucleotide polymorphism arrays (SNP-arrays) allow the entire genome to be screened for copy-number alterations (CNAs) in a single experiment. In contrast to CBA analyses, input of array platforms only includes DNA and obviates the need for fresh isolated tumor cells and in vitro mitogen stimulation.^16–18^

An overview of the main limitations/benefits of CBA, FISH and CMA in CLL^19^ is provided in Table 1.

**Table 1.** Comparison of CBA, FISH, and CMA for the Detection of Genomic Changes in CLL

Cytogenetic test	Advantage (strength) of the test	Disadvantage (weakness) of the test
CBA	Genome-wide scan Single cell analysis Detection of balanced chromosome rearrangements (ie, translocations, inversions) Sensitivity is circa 10% to 15% in routine analysis: 2 (loss) or 3 (gain) aberrant metaphases found in 20 analyzed cells Detection of clonal evolution Discovery of novel abnormalities (and complex karyotype)	Requires culturing of cells with B-cell mitogen (eg, IL2 + CpG) to increase sensitivity Resolution limit is 10–20 Mb Exact definition of rearrangements may not be evident by banding-analysis alone Cannot detect regions of homozygosity (CN-LOH) Analysis is laborious and slow (1 case at a time)
FISH	Resolution is circa 150–900 kb, depending on probe-size Sensitivity for detection of low level clones is around 3%–5% (usually 100–200 interphase nuclei scored) Does not require cultured cells Batch cases	Detects only abnormalities where the probe was designed for Multiple FISH probes are required to look at diverse abnormalities (routinely in CLL a 4–5 probe panel) Clonal evolution may be overlooked Cannot detect regions of homozygosity (CN-LOH) or genomic instability (chromothrypsis)
CMA	Whole genome scan Resolution is 50 kb or less (depending on platform design) Discovery of novel abnormalities (and genomic complexity) Detection of regions of homozygosity (CN-LOH) if SNP-based platform Significantly automated (batch cases) Does not require cultured cells Discovery of novel unbalanced abnormalities with exact definition of the regions (and genes) involved (within the limit of resolution) Detection of (submicroscopic) regions with genomic instability/chromothripsis	Cannot detect balanced chromosome rearrangements Detection of multiple clones is feasible but not evident Sensitivity is 10%–20% (platform-dependent) B-cell enrichment may be required if tumor burden is low

CBA = chromosome banding analysis; CLL = chronic lymphocytic leukemia; CMA = chromosome microarray analysis; CN-LOH = copy neutral loss of heterozygosity; FISH = fluorescence in situ hybridization.

Adapted from Cooley et al, Schoumans et al, and Chun et al.^18,40,42^

### Summary statement

Cytogenetic analysis in CLL is feasible and can be performed with various methodologies (FISH/CBA/CMA). Each one of them provides different but complementary information regarding the genomic background of the malignant clone.

## METHODOLOGICAL ISSUES FOR CBA

### Samples

For the great majority of patients with CLL, peripheral blood (PB) is the most appropriate source of tumor cells as it usually has a high CLL cell fraction. Bone marrow (BM) samples may have higher amounts of contaminating nonmalignant cells; on the other hand, they are especially suitable for cytogenetic cultures in cases with few circulating clonal cells. Finally, lymph node biopsies may be an alternative option to obtain tumor cells, that is, in small lymphocytic lymphoma.

A total of 5 to 15 mL of PB (or alternatively 1 to 2 mL of BM) should be collected in heparinized tubes. Ethylenediaminetetraacetic acid (EDTA) is not suitable for cytogenetic cultures. That said, based on our experience, if the sample arrives in EDTA and it has been in this anticoagulant for <12 hours, the sample can still be washed twice with sterile Roswell Park Memorial Institute (RPMI) medium or 10× phosphate-buffered saline (PBS) before setting up cultures, especially if there is no possibility of obtaining a new heparinized sample. As a general recommendation, optimal results are obtained within 24 hours after collecting the sample. The transport time is highly relevant for samples with high white blood cell (WBC) counts.^20–22^

### Methods

Before establishing cell cultures, the WBC count from the sample must be determined. A total of 2 × 10^6^ leukocytes/mL medium will be added to culture tubes or flasks.^20^ As mentioned above, addition of mitogens to the media is essential. This is due to the fact that regulation of early cell cycle progression differs between CLL cells versus normal B cells: indeed, the former are mostly arrested in the G_0_/early G_1_ phase of the cell cycle and characterized by marked hyporesponsiveness towards a variety of polyclonal B-cell activators.

From a practical point of view, it is recommended to set up 2 parallel cultures with different cell mitogens for each patient, one with 12-*O*-tetradecanoly-phorpol-13-acetate and the other with IL-2 plus DSP30. Following this strategy, more cases with abnormal karyotypes are identified, as 5% to 20% of cases are found carrying aberration(s) only with 1 of the 2 mitogens. CLL cells remain in culture for 72 hours and, after that, the antimitotic colcemid is added to the media to obtain metaphases (Table 2).

**Table 2.** Technical Recommendations for Chromosome Banding Analysis in CLL

Item	Recommendations	Remarks
Materials		
Anticoagulants	Heparin	EDTA is not suitable for cytogenetic cultures. However, in such case, it is worth trying to wash the sample twice with sterile RPMI medium or 10× PBS and set up cultures (if there is no other option)
Cells/tissue	Peripheral blood	Bone marrow or lymph node biopsies may be an option in those cases with few circulating clonal cells
Conditions	Set up cultures before 24 h sample obtention	The transport time is highly relevant for samples with high WBC counts 20–22
Methods^*a*^		
WBC count	Adjust cultures to 2 × 10^6^ leucocytes/mL medium	
Mitogens	Set up 2 parallel cultures: Culture A: 50 µL TPA Culture B: 500 µL IL2 + 100 µL (10 nmol) DSP30^*b*^	Following this strategy, more cases with abnormal karyotypes are identified, as 5% to 20% of cases are found carrying aberration(s) only with 1 of the 2 mitogens^8^
Culture times and Colcemid^*c*^	Culture A: incubate 72 h at 37°C, add 50 µL Colcemid, incubate 2 h, harvest Culture B: incubate 48 h at 37°C, add 100 µL Colcemid, incubate 16–24 h, harvest	
Analysis		
Number of metaphases	20, fully analyzed	Recommended to avoid overlooking subclonal aberrations and complexity underestimation^49^
Interpretation-clinical significance		HC (≥5 structural/numerical abnormalities in the same clone) is generally associated with unfavorable prognosis, excepting patients with a CK harboring +12, +19. The predictive significance of high-CK in the era of novel agents is still unclear

^*a*^Harvesting, slides preparation, aging, and staining are performed following standard cytogenetic procedures.^50^

^*b*^DSP30: sequence: 5′-TsCsgsTsCsgsCsTsgsTsCsTsCsCsgsCsTsTsCsTsTsCsTsTsgsCsC.

^*c*^Colcemid concentration 0.15 g/mL.

CK = complex karyotype; CLL = chronic lymphocytic leukemia; EDTA = ethylenediaminetetraacetic acid; HC = high-complexity; PBS = phosphate-buffered saline; RPMI = Roswell Park Memorial Institute; WBC = white blood cell; TPA = 12-*O*-tetradecanoly-phorpol-13-acetate.

After incubation, harvesting of the cultures is performed following standard cytogenetic procedures.^21,22^ This can be undertaken manually or using automated robots. Basically, after centrifugation and removal of the supernatant, the cellular sediment is resuspended in potassium chloride hypotonic solution and incubated for 10 minutes at 37°C. The cell suspension is centrifuged again, supernatant is discarded and cells are fixed with fresh prepared Carnoy’s solution (3 methanol/1 acetic acid). After centrifugation and removal of supernatant, the sediment is resuspended again with Carnoy’s solution. This process is repeated 2 to 3 times until the pellet is clean (yellow/white). Finally, a cell suspension is obtained, adjusted at an optimal cell concentration (according to turbidity of the sample), and slides are prepared with 1 to 2 drops. It is important to dry the slides correctly to obtain good quality banding and staining. Once the slides are prepared, an aging process is required. After that, banding and staining is carried out using Giemsa or Wright’s solution, after 2× saline-sodium citrate or trypsin incubation, to obtain chromosome banding. In most cases, the resolution will be of 200 to 400 bands per haploid karyotype.

### Evaluation of metaphases

Metaphases should be screened at the microscope or captured using a metaphase finder. A minimum of 20 metaphases should be analyzed in cases with normal karyotype. Ten metaphases should be fully analyzed, with a further 10 analyzed or counted and scored for relevant structurally abnormal chromosomes.^11^

### Summary statement

Standardized methodologies should be followed when performing CBA in CLL. These are summarized in Table 2.

## BIOLOGICAL CONSIDERATIONS AND CLINICAL IMPLICATIONS: FOCUS ON CYTOGENETIC COMPLEXITY

### Cytogenetic complexity in hematological malignancies

Cytogenetic complexity is found in many types of tumors and can arise through multiple mechanisms. The combination of cytogenetic complexity and underlying genomic instability results in altered expression potentially of large numbers of genes and in genomic diversity, thereby enabling tumors to respond rapidly to selective pressures.^23^

In hematological malignancies such as myelodyspastic syndromes, acute myeloid leukemia, and acute lymphoblastic leukemia, a CK has independent prognostic and/or predictive value and the number of abnormalities with the greatest prognostic significance varies between 3 and 5 per clone.^24,25^ This variability in the clinical significance of CK reflects its underlying biological heterogeneity such that a clinically relevant definition is only possible within the context of an individual disease and should also take into account other features that influence cellular phenotype, including specific chromosomal abnormalities, gene mutations, the cell of origin, and microenvironmental interactions as well as specific treatment modalities.

### Cytogenetic complexity in CLL

In CLL, CK is classically defined as the presence of ≥3 clonal structural or numerical abnormalities. Although present in 8% of monoclonal B lymphocytosis,^26^ CK ≥3 is associated with advanced stage disease, cases harboring unmutated IGHV genes (U-CLL), del(11q), *TP53* aberrations [del(17p) and/or *TP53* mutation], and telomere dysfunction.^26–28^

There is both experimental and in vivo data to indicate that (at least) some of the aforementioned associations are causal.^9,26,28^ *TP53* aberrations promote genomic instability through a variety of mechanisms and also enable cells to tolerate the proteomic, metabolic, and other cellular stresses which arise secondary to major structural abnormalities.^29^

That said, not all CKs arise in synergy with *TP53* dysfunction. It has been recently reported that in almost 20% of patients with CK in CLL, even among those with more than 5 chromosomal aberrations (high CK), no *TP53* aberration could be detected despite using high-sensitive detection methods, that is, targeted next generation sequencing.^26^ Whether CK in these cases is an independent biological phenomenon or closely related to a non-p53 dependent, still undefined, pathophysiologic mechanism, needs to be further investigated.

Genomic aberrations have traditionally been considered as a clonal signature of malignant cells. The situation appears more complex in mature B cell malignancies, including CLL, where the landscape of genomic aberrations is heterogeneous and diverse even within the same case: hence, such aberrations most often define subclones rather than the clone in its entirety.^12^ Contrasting this intraclonal genomic heterogeneity, all CLL clonal cells express an identical B cell receptor immunoglobulin (BcR IG) whose unique features critically impact on the natural history of CLL.^30^

With this in mind, it is indeed remarkable that specific genomic aberrations are significantly enriched in CLL subgroups defined by the expression of BcR IG with distinct immunogenetic features, suggesting links between particular antigenic triggering and distinct pathways of genomic evolution.^31^ CK fits with this pattern, considering that more than 75% of high-CK cases concern U-CLL, likely reflecting their high proliferative capacity.^26^ At the other end of the immunogenetic spectrum, it is worth mentioning that ~25% of the (infrequent) CLL cases with mutated IGHV genes (M-CLL) displaying CK are classified in a distinct subgroup carrying co-existing trisomies of chromosomes 12 and 19 along with other numerical (usually +18) and/or structural abnormalities (see below).

In CLL, intense cellular proliferation has been associated with extensive telomere shortening which can be detected even in early stage CLL and is a powerful independent marker of poor outcome.^28,32^ Telomere dysfunction leads to telomere fusion with both telomeric and non-telomeric loci, formation of dicentric chromosomes, initiation of breakage–fusion–bridge cycles resulting in a variety of structural abnormalities which include deletions, duplications, non-reciprocal translocations, and chromothripsis and may also drive genomic instability, facilitating selection of clones with del(17p) or del(11q).^27,28^

#### Heterogeneity within CLL cases with CK

i. Number of abnormalities: The prognostic significance of the number of chromosomal abnormalities in cases with CK ≥3 was first observed in studies of factors influencing time-to-first-treatment^33^ and outcome following allogeneic stem cell transplantation.^34^ In each study, CK ≥5 was associated with a poorer outcome. The recent retrospective ERIC study of 5290 patients managed during the era of chemo(immuno)therapy enabled a more refined analysis of the clinicobiological associations and clinical impact of CK in CLL. CK cases were subdivided into 3 subgroups based on whether they were carrying 3, 4, or ≥5 abnormalities (comprising 45%, 21%, and 34% of all CK cases, respectively). Patients with ≥5 abnormalities, defined as high-CK, had a very poor outcome (median overall survival [OS] of 3.1 years) independently of clinical stage, *TP53* aberrations, and IGHV gene somatic hypermutation status. In contrast, CK cases with 3 or 4 aberrations (low-CK and intermediate-CK, respectively) had a shorter survival (median OS of 4.3 years) only when accompanied by *TP53* aberrations.^26^ These findings appear relevant for risk stratification of patients with CLL. However, caution is still warranted since the published evidence derives from retrospective studies mostly performed outside clinical trials with all the implicit caveats.
ii. Type of abnormalities
  Multiple trisomies: Approximately 10% of cases with trisomy 12 also have trisomy 19 and are characterized by a distinctive constellation of features, including mutated IGHV genes, biased expression of lambda light chains, exclusive expression of IgG-switched heavy chains, a low prevalence of TP53 aberrations and a more indolent clinical course than cases with isolated trisomy 12. The great majority (~70%) of +12, +19 cases also display additional trisomies, usually +18, and/or structural abnormalities, most frequently del(13q), thereby fulfilling the criteria for CK.^35^ Such cases comprise 10% of all CK ≥3 cases and are characterized by an extremely indolent course with prolonged time to first treatment (TTFT) and OS which is longer than either other CK cases or cases without CK, including other M-CLL.^26,33^
  Specific copy number aberrations: In patients with CK, especially those with CK ≥5, the distribution of structural abnormalities not detected by the standard FISH panel is non-random. Many studies employing CBA or genomic arrays have identified recurring gains of 2p, 3q, 8q, and losses of 3p, 4p, 6q, 8p, 9p, 15q, and 18p, many of which encompass genes known to be relevant to CLL biology. In univariate analyses, gains of 2p and 8q and losses of 9p and 18p in unselected cohorts, and gains of 8q and losses of 3p, 8p and 9p in cases with del(17p) (especially if due to i(17q)) have been associated with poor outcome in the chemo/chemoimmunotherapy era,^9,13,36^ while resistance to ibrutinib has been associated with loss of 8p and 18p.^37^ However, larger cohorts will be required to demonstrate independent prognostic significance of specific copy number aberrations in multivariable analyses which include CK ≥5.
iii. Presence of subclones: CKs may show considerable karyotypic heterogeneity. In one study, the median number of clonal aberrations was 7 (range 3 to 17) and clonal evolution or a composite karyotype (when clonal heterogeneity was too complex to allow enumeration of individual subclones) was found in 74% of patients.^32^ The potential clinical significance of measuring clonal heterogeneity is supported by preliminary results using a new method for inferring clonal heterogeneity from SNP array data. When applied to samples from 258 previously untreated CLL patients, 44% had >1 clone with a maximum of 3 clones. The presence of multiple clones was significantly associated with Rai stage, age at diagnosis, del(17p), and del(11q). There was a statistically significant independent association between the presence of multiple clones and OS even when accounting for CK ≥3 as a potential confounder.^38^ Of note, molecular methodologies such as short read whole genome sequencing (WGS) or CMA are unable to identify the different independent clones and therefore cannot provide information on the intraclonal cytogenetic heterogeneity.

### Summary statement

The well-known heterogeneity of CLL extends even within patients with cytogenetic complexity, who should not be considered a priori equivalent.

### Chromosome microarray analysis

CMA was initially developed as a genetic discovery tool in research laboratories in the 1990s. A decade later, this was moving rapidly into the clinical cytogenetic laboratories for the evaluation of constitutional chromosomal aberrations as well as acquired genomic abnormalities in human cancers, including hematological malignancies such as CLL.

Nowadays, most cytogenetic laboratories apply Agilent, Affymetrix, or Illumina platforms for CMA in hematological malignancies.^39^ The Illumina and Affymetrix SNP-arrays effectively combine the detection of CNAs and copy-neutral events (CN-LOH) with very high resolution.

### Analysis and interpretation of microarray data in CLL

Tools for analysis and criteria for interpretation and reporting of microarray-based genomic profiling have been previously provided.^17,18,40^ Cytogenetic laboratories have primarily used these guidelines in CLL diagnostics. In brief, interpretation criteria are set in such a way that non–tumor-related copy number changes are excluded. Only gross CNAs (≥5 Mb) and CN-LOH (≥10 Mb and extending to the telomeres) are considered as tumor-associated abnormalities. Focal CNAs (<5 Mb) are only reported when they involve known tumor-related genes (https://cancer.sanger.ac.uk/cosmic).

### Clinical use of chromosomal microarray analysis in CLL

Microarrays are well suited for genome-wide analysis in CLL resulting in accurate detection of chromosome abnormalities with established prognostic value in CLL (trisomy 12, deletions of 13q14, deletions of 11q22, and deletions of 17p13) but also allowing for the discovery of genomic complexity (GC). That said, small clones may be overlooked. The presence of 3 or more genomic abnormalities is signified as GC and is associated with shorter survival and advanced disease.^36,41^ Many relapsed/refractory CLL cases display GC with additional CNAs next to the high-risk *TP53* or *ATM* deletions. Chun et al^42^ summarized a list of chromosome regions (and probably involved genes) repeatedly observed in complex array profiles in patients with CLL. More recently, Leeksma et al^36^ “re-defined” GC in a large, retrospective ERIC CLL-cohort studied by CMA, and subdivided GC in low-GC (0 to 2 CNAs), intermediate-GC (3 to 4 CNAs) and high-GC, respectively (≥5 CNAs), reporting overall similar profiles with Chun et al.^42^ Very similar to CBA analysis, only high GC defined as ≥5 CNAs emerged as an independent adverse prognosticator on multivariable analysis for TTFT (hazard ratio: 2.15) and OS (hazard ratio: 2.54).^36^

In the past, laboratories have been struggling to find a comprehensive and clear way of interpreting acquired genomic abnormalities by CMA and this has certainly hindered the application of microarray analysis in clinical trials, including those in CLL. Chun et al^42^ have provided best practice tools for standardization giving weight to the clinical impact of CMA-detected changes on medical care (diagnostic, prognostic, and therapeutic significance). Applying these standards, CMA is well suited for accurate genome-wide analysis in CLL at a resolution higher than those of CBA and FISH. This is clinically relevant, considering that CK has been demonstrated as an independent negative prognostic factor in CLL and should be taken into account in clinical studies using novel agents.^43^

### Summary statement

CMA is a promising methodology for the identification of GC in CLL.

## INTERPRETATION OF CYTOGENETIC FINDINGS IN THE CLINICAL SETTING

Based on a number of prospective clinical trials, the latest iwCLL guidelines for the management of CLL recommend performing FISH analysis as well as analysis of *TP53* gene in all patients with CLL, in both general practice and clinical trials. In contrast, due to scarce evidence from prospective clinical trials, iwCLL recommends the use of CBA only in the context of clinical trials, rather than the routine clinical setting.^44^ This recommendation is mostly based on recent reports highlighting the prognostic significance of CK^26,45^ which, presently, can be detected only through CBA, as suggested by the current iwCLL guidelines.^44^ Against this background, no concrete recommendations are provided on how to integrate and interpret the CBA findings toward more refined clinical decision making, in particular for the treatment choice. The same guidelines recommend performing FISH in the context of both clinical practice and clinical trials, while CMA is not generally recommended. That said, other consortia have recently included CBA in the diagnostic work-up of CLL.^46,47^

Reports suggest that CK is associated with clonal evolution and chemorefractoriness.^48^ However, these reports are mostly based on retrospective studies since for many years CBA was not considered a standard diagnostic method in CLL and, therefore, not systematically assessed even in the context of clinical trials. Hence, unsurprisingly, the potential predictive value of CK, especially in the era of novel targeted agents, remains to be conclusively determined. The lack of standard methodology, the clinico-biological heterogeneity of the studied cohorts, the low number of patients carrying CK as well as the current practice of considering all CKs as a homogeneous group may explain the discrepancies between different studies.^49–51^

Regarding CMA, most published data have been obtained retrospectively from real-world databases, exhibiting largely concordant results when compared to CBA studies.^16,41^ That said, high GC (≥5 CNAs) has been recently associated with dismal clinical outcome also in a clinical trial context (MURANO trial) in patients treated with both standard chemoimmunotherapy (bendamustine + R) and novel agents (BCL-2 inhibitor venetoclax + rituximab).^43^

Presently, CBA is the only standardized methodology that is capable of providing information of the whole genome, while also allowing the overview of the clonal landscape and intraclonal hierarchy. Novel methodologies such us WGS appear promising. However, despite the advances regarding the technical standardization several issues remain open particularly regarding the harmonization of the interpretation of the WGS findings in the clinical setting and whether it can replace CBA without the need of validation. CBA offers the possibility to detect chromosomal aberrations not covered by the standard FISH panels/probes and also reveals the existence of CK which is not possible using standard FISH-based diagnostics. Still, it is not yet precisely known how to use CBA information in the clinical setting and open questions abound. Indicatively, should all CKs be considered as equivalent? Should chemoimmunotherapy regimens be avoided in patients with CK and, if yes, which should be the recommended treatment? Should the search for cytogenetic complexity by CBA be undertaken in all patients before treatment or only restricted to cases lacking *TP53* aberrations? Besides the aberrations included in the standard FISH-analysis, are there additional recurrent cytogenetic abnormalities with prognostic or predictive significance in CLL, especially in the era of the novel agents? If access to a robust methodology is available, when should CBA be performed in CLL? How should CBA results be reported and interpreted in CLL? Could CBA be replaced by a less cumbersome method?

Starting from the final question, despite recent advances in next generation sequencing, the time is not yet right for giving up CBA in the routine diagnostic setting. WGS may appear as an attractive future alternative, however several issues, including both high cost and the lack of methodological standardization and harmonization, preclude considering it as a realistic imminent option. As mentioned above, CMA could serve as a surrogate methodology, at least for the identification of cases with high GC.^36^ That said, even within this group of patients there is significant discordance between CBA and CMA.^19^

**Figure 1.**
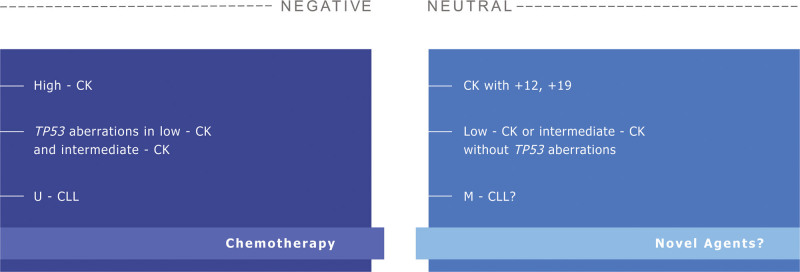
**The clinical significance of complex karyotype in CLL.** Disease features as well as different treatment options may either aggravate the negative impact of complex karyotype (listed in the dark blue panel at the left) or have a neutral effect (listed in the light blue panel at the right). CK = complex karyotype; CLL = chronic lymphocytic leukemia.

Since the precise significance of CK in CLL is still debatable, ERIC holds the view that the most prudent policy is “watch and wait” until the evidence is substantial and robust enough for allowing safe recommendations. We therefore encourage performing CBA before treatment administration but dissuade from using only CBA findings for treatment decision making. Identification of CK seems highly relevant clinically but caution is needed to avoid overdiagnosis. In the absence of *TP53* aberrations, low and intermediate CK (3 and 4 chromosomal aberrations, respectively) should not be considered by default as a synonym for aggressive disease. Moreover, in line with the notion that cut-offs in medicine are often arbitrary, we should refrain from considering all CKs as equivalent, a view amply exemplified by the case of CK with +12, +19 (Figure 1). Finally, we strongly recommend to always perform CBA within clinical studies and endorse the inclusion of patients with CK or other cytogenetic abnormalities of potential clinical significance in clinical trials with novel agents.

Regarding CMA, we definitely encourage its inclusion in the context of clinical trials, however not yet in routine clinical practice. More generally, we would also like to underscore the fact that, despite being capable to detect recurrent aberrations that dictate treatment choices, CMA is less appropriate than CBA for the detection of CK, particularly due to its inherent limitation to detect structural aberrations (ie, translocations), although, admittedly, it may be informative for the grade of GC.^19^ Evidently, therefore, more data are needed to reach definitive conclusions.

## CONCLUSION

Mounting evidence suggests that CBA has the potential of becoming a useful diagnostic tool in CLL, especially as it is standardized end-to-end. However, the clinical significance of the CBA findings is not yet fully elucidated, especially regarding CK but also other cytogenetic abnormalities not included in the standard CLL FISH-panels. The same stands for CG detected by CMA. To obtain definitive insight into the applicability/relevance of CBA findings in the routine clinical setting, we recommend to systematically perform CBA as a standard assessment in the context of prospective clinical trials. If available, CMA could also be performed either as a complement to CBA or as a sole methodology when CBA is not feasible. On the other hand, we dissuade from using only CBA/CMA findings for treatment decision making since the predictive value of cytogenetic findings such as CK is still debatable, especially in the era of targeted treatments.

## AUTHOR CONTRIBUTIONS

PB, BE, CM, APK, DO, CH, and KS conceived the idea, analyzed data, and wrote the manuscript. MJ, PG, and AA made a critical review of the document. All authors read and approved the final version of the manuscript.

## DISCLOSURES

PG is a HemaSphere Editor. The remaining authors have no conflicts of interest to disclose.

## SOURCES OF FUNDING

Supported in part by the Hellenic Precision Medicine Network in Oncology; MJ CZ - DRO (FNBr, 65269705), the research projects MZ ČR AZV NV19-03-00091, NU20-08-00314 and MZ ČR AZV NU21-08-00237; Lion’s Cancer Research Foundation, Uppsala and Generalitat de Catalunya (17SGR437).
